# Rapid Rule-Based Reward Reversal and the Lateral Orbitofrontal Cortex

**DOI:** 10.1093/texcom/tgaa087

**Published:** 2020-11-17

**Authors:** Edmund T Rolls, Deniz Vatansever, Yuzhu Li, Wei Cheng, Jianfeng Feng

**Affiliations:** 1 Institute of Science and Technology for Brain-inspired Intelligence, Fudan University, Shanghai, 200433, China; 2 Oxford Centre for Computational Neuroscience, Oxford, UK; 3 Department of Computer Science, University of Warwick, Coventry CV4 7AL, UK

**Keywords:** anterior cingulate cortex, depression, emotion, non-reward, orbitofrontal cortex, reversal, reward

## Abstract

Humans and other primates can reverse their choice of stimuli in one trial when the rewards delivered by the stimuli change or reverse. Rapidly changing our behavior when the rewards change is important for many types of behavior, including emotional and social behavior. It is shown in a one-trial rule-based Go-NoGo deterministic visual discrimination reversal task to obtain points, that the human right lateral orbitofrontal cortex and adjoining inferior frontal gyrus is activated on reversal trials, when an expected reward is not obtained, and the non-reward allows the human to switch choices based on a rule. This reward reversal goes beyond model-free reinforcement learning. This functionality of the right lateral orbitofrontal cortex shown here in very rapid, one-trial, rule-based changes in human behavior when a reward is not received is related to the emotional and social changes that follow orbitofrontal cortex damage, and to depression in which this non-reward system is oversensitive and over-connected.

## Introduction

The human orbitofrontal cortex is a key brain region involved in emotion, and this is related in part to its roles in representing reward (Rolls 2014, 2019b, 2019c; Rolls et al. 2020b). However, not only is reward represented in the human medial and mid-orbitofrontal cortex, but aversive, unpleasant, stimuli are represented, especially in the lateral orbitofrontal cortex (Grabenhorst and Rolls 2011; Rolls 2019b). In the research described here, we show that when behavior must change very rapidly, in one trial, because a reward has not been obtained, the lateral orbitofrontal cortex is activated. This specialization of the human lateral orbitofrontal cortex is fundamental to understanding many aspects of human social and emotional behavior and is important in understanding disorders of emotion such as depression, as described here. The type of reward reversal investigated here is key in understanding the human orbitofrontal cortex, because it is performed in one trial which indicates great flexibility of reward-related behavior, is non-associative, cannot be accounted for by model-free reinforcement learning, and represents a primate specialization that cannot be performed by rodents. It is shown that in this reward reversal task the human right lateral orbitofrontal cortex and adjoining inferior frontal gyrus is activated. These regions are implicated causally in the reversal by previous findings showing that a similar non-probabilistic reward reversal task is impaired (indicated by a failure to reverse), in humans with damage to the orbitofrontal cortex (Rolls et al. 1994).

A key computation performed by the primate including human orbitofrontal cortex is rapid re-learning about the reward value of stimuli, which is impaired by orbitofrontal cortex damage in macaques (Butter 1969; Iversen and Mishkin 1970) and humans (Rolls et al. 1994; Fellows and Farah 2003; Berlin et al. 2004; Hornak et al. 2004; Fellows 2011). The reward reversal learning is rapid, in that it can occur in one trial, as follows (Thorpe et al. (1983). Assume that visual stimulus 1 is associated with reward and a response can be made to obtain the reward, and visual stimulus 2 is associated with punishment so that no response should be made to it, in a Go-NoGo task. If the reward contingency is then suddenly reversed, so that a response to stimulus 1 previously associated with reward now receives punishment, then on the very next trial on which stimulus 2 is shown, participants choose stimulus 2, even though its previous association was with punishment or loss. This type of reward reversal must thus be based on application of a rule, which must be held in memory, about which stimulus is currently associated with reward, and that if unexpectedly reward is not obtained, then behavior should change, and the other stimulus must now be chosen to obtain reward. This rapid one-trial reward-based reversal is learned over a number of such reversals, and is called reversal learning set. This non-associative one-trial rule-based reward reversal was discovered to be represented in the responses of single neurons in the macaque orbitofrontal cortex (Thorpe et al. (1983), and because the reversal occurred in one trial, it must have been non-associative, and therefore rule-based or model-based. This concept of rule-based rather than purely associative mechanisms for reversal was formalized in a biologically plausible model of rule-based reward reversal (Deco and Rolls 2005). This one-trial rule-based reversal cannot be accounted for by model-free reinforcement learning (Schultz 2016; O'Doherty et al. 2017; Schultz 2017), as described below, and requires a model that if a reward is not obtained, behavior should change to a different stimulus, even if its recent reward history is non-reward (Deco and Rolls 2005). Model-based approaches to reward reversal have been used in a number of subsequent investigations of the orbitofrontal cortex (Wilson et al. 2014; Schuck et al. 2016; Wang et al. 2020).

This rule-based reversal learning occurs in primates including macaques and humans and is likely to be very adaptive in social and related behavior, in that at the slightest indication that an individual’s behavior is no longer receiving reward, then it can change immediately. For example, a slight frown or change of facial expression from someone with whom one is in conversation might lead one to understand that the subject of the conversation should change. This rapid one-trial reversal does not happen in rodents (Hervig et al. 2020) and may be a key specialization of the primate including human orbitofrontal cortex that enables rapid reciprocation and changes of behavior in social interactions (Rolls 2019b). Understanding how this rapid reward-based reversal is implemented is thus likely to be important in understanding human emotional and social behavior, and their disorders.

We emphasize that this type of reward learning is very different from model-free reinforcement learning involving reward prediction errors (Schultz 2016; O'Doherty et al. 2017; Schultz 2017), which is typically slow and involves probabilistic tasks (Rolls 2021a). Model-free reinforcement learning is not only slow but has no mechanism for learning to switch the rule about which stimulus is currently rewarded. In this context, the rapid reward value reversal investigated here is key in the reward-related functioning of the primate including human orbitofrontal cortex (Rolls 2019b, 2021a). In more complex paradigms, reward value reversal of the type investigated here may be measured by tasks involving what has been termed intradimensional shift (Pantelis et al. 1999).

It has been shown that there are neurons in the macaque orbitofrontal cortex that in this Go-NoGo visual reward reversal task reverse the stimulus to which they respond in the rule-based, non-associative, way just described (Thorpe et al. 1983; Rolls et al. 1996). These neurons respond to the expected reward value of a stimulus, and other single neurons respond to the expected punishment value of a stimulus. Further, in this one-trial rule-based visual discrimination reversal, there is a different population of macaque orbitofrontal cortex neurons that respond only in reversal, when the expected reward is not obtained, and reversal of the reward value should occur (Thorpe et al. 1983). These neurons have been described as “non-reward” neurons, and similar neurons have been described by others (Rosenkilde et al. 1981). These neurons reflect errors made when the reward value of stimuli needs to be reversed, in that representations of the reward value of stimuli are found in the orbitofrontal cortex, but behavioral responses or actions are not represented (Thorpe et al. 1983; Rolls et al. 1996; Wallis and Miller 2003; Padoa-Schioppa and Assad 2006; Grattan and Glimcher 2014; Rolls 2019b). We emphasize that the learning investigated here is about the reward value of stimuli, not of actions.

It is important to understand this reward reversal learning, and neural responses to non-reward, better in humans, partly because the orbitofrontal cortex is implicated in emotional disorders in which there is altered sensitivity to non-reward as described above, and in depression, which may involve responses to non-reward that produce sadness (Rolls 2016c, 2018, 2019c). In economic decision-making for monetary rewards, it has been shown that the medial orbitofrontal cortex is activated by monetary reward, and the lateral orbitofrontal cortex by losing money (O'Doherty et al. 2001; Xie et al. 2020). The orbitofrontal cortex is implicated in the Iowa gambling task (Glascher et al. 2012). However, these tasks involve probabilistic delivery of rewards or losses for stimuli and so do not directly assess the type of one-trial reward-based reversal learning in which the primate orbitofrontal cortex is implicated by the lesion evidence described above (Rolls 2019b).

In the present investigation, we therefore measured brain activations in the deterministic one-trial rule-based reward reversal task described above, to assess the roles of different parts of the orbitofrontal cortex and other brain regions to the non-reward signaling involved in one-trial reward value reversal. The task design was a Go-NoGo visual discrimination reversal task, specially implemented to allow direct comparison with neuron-level findings in this particular task in the orbitofrontal cortex (Thorpe et al. 1983; Rolls et al. 1996), ventral striatum (Williams et al. 1993), basal forebrain (Wilson and Rolls 1990a, 1990b), amygdala (Sanghera et al. 1979), and inferior temporal visual cortex (Rolls et al. 1977). The design of this task also enables activation related to winning points, and to losing points, to be measured.

To our knowledge, this is the first time that a deterministic one-trial reversal-learning task involving simple rewards (points in humans) has been investigated with neuroimaging in humans. In a more complex and probabilistic task involving face expressions provided for particular individual faces, activation during reversal was found in the lateral orbitofrontal cortex and supracallosal anterior cingulate cortex, but the task design did not allow reward and loss representations to be assessed (Kringelbach and Rolls 2003). In macaques, the neuroimaging evidence available is for a reversal learning task though not for one-trial rule-based reversal, and evidence was found that the lateral orbitofrontal cortex was activated when the behavior had to change (Chau et al. 2015).

Given the above, the aims of the investigation were to analyze how different brain regions are involved in one-trial rule-based visual discrimination reversal, including measuring activations on the reversal trials in which not obtaining an expected reward must be used to reset the rule for which stimulus is currently rewarded; and measuring the activations to winning (on Go trials when 25 points was won), and to losing (on NoGo trials when 5 points were lost). The results described here focus on anterior brain regions including the orbitofrontal cortex, cingulate cortex, amygdala, and insula, as these brain areas are implicated in reward and non-reward as shown by the effects of brain damage to these regions (Rolls et al. 1994; Berlin et al. 2004; Hornak et al. 2004; Fellows 2011; Rolls 2019b, 2019c, 2019a, 2021a).

## Methods

### The Go-NoGo Visual Discrimination Reward Reversal Task

**Figure 1:**
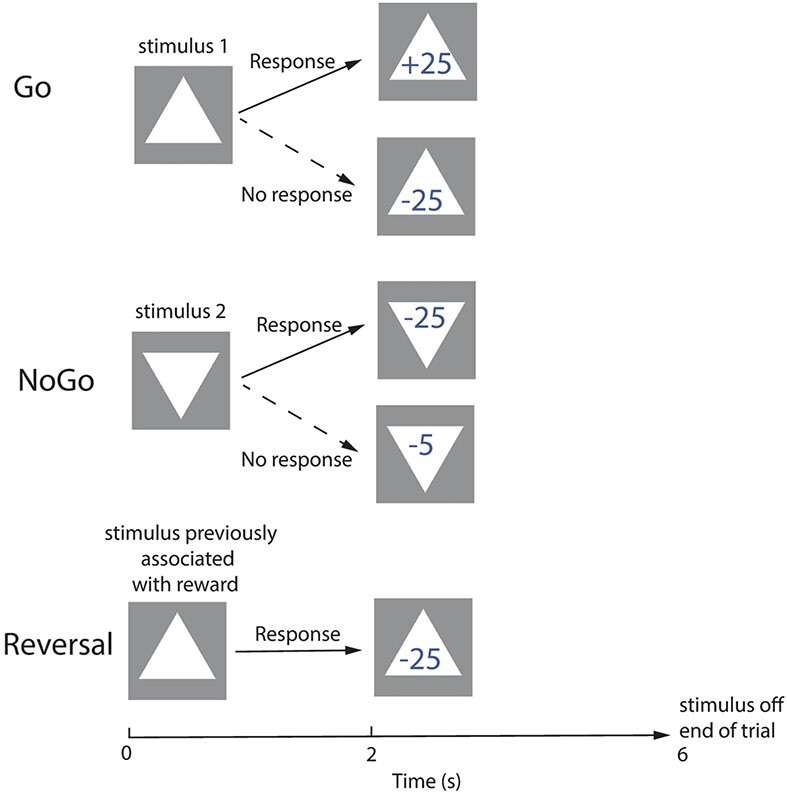
The Go-NoGo rule-based one-trial visual discrimination reversal task. On a Go trial, when the visual stimulus recently associated with reward was presented, if the participant pressed the response button within 2 s, 25 points were obtained. A failure to respond resulted in the loss of 25 points. On a NoGo trial, when the stimulus recently associated with a loss was presented, the participant lost 25 points if a response was made. If, correctly, no response was made, the participant lost only 5 points. On a reversal trial presented at a random point in the sequence, when the visual stimulus recently associated with reward was shown, the participants responded expecting reward, but lost 25 points, and on the very next trial the participants treated each of the two visual stimuli according to the opposite rule for which stimulus would be rewarded, as a result of pretraining experience with the task. As described in the Methods, two types of Control trial were included in the trial sequence randomly: on a response control trial when a circle was shown the participant had to press the button, and on a NoResponse trial when a square was shown the participant had not to respond, but the Outcome in both cases was 0 points, so that neither control stimulus was associated with reward. There were 100 pseudo-randomized trials (see Methods).

This was a deterministic task with two visual stimuli, one of which was associated at any one time with a win of 25 points if a response was made to it, and the other with a loss of 5 points. One stimulus was presented on each trial, preceded by a 0.5-s fixation cross to enable the subject to be ready before the stimulus appeared. The discriminative stimuli were a triangle and an inverted triangle (see Fig. 1). The task was designed to be similar to that used in complementary primate single neuron neurophysiological investigations (Thorpe et al. 1983; Rolls et al. 1996), and in patients with damage to the orbitofrontal cortex (Rolls et al. 1994).

On a Go trial, the stimulus currently associated with reward was presented, and if the participant pressed the response button within 2 s, the number “+25” appeared on top of the stimulus at the end of the 2-s period and remained on during this outcome part of the trial until the stimulus was turned off after 4 s. If the participant failed to respond, “−25” was shown in the outcome phase.

On a NoGo trial, the non-reward stimulus of the pair was shown, and if the subject responded, a loss of 25 points was shown in the outcome phase. If the participant correctly did not respond, then the loss was only 5 points.

On a reversal trial, the currently rewarded stimulus was shown, and when the participant responded, a loss showing as −25 was shown on top of the stimulus in the 4-s outcome phase. This indicated to a participant that a reversal of the contingencies must have taken place.

The participants made few errors in the task, and all learned to reverse in one trial with rule-based reversal. That is, after a reversal trial, on the very next trial they treated the stimuli in the opposite way to before the reversal, for example, by responding to the previously non-rewarded stimulus, and not responding to the pre-reversal rewarded stimulus. The trial order was pseudo-randomized with equal numbers of Go and NoGo trials in every 10 trials, and with reversal occurring at a random number of trials between 8 and 12 trials after the previous reversal.

Two types of control trials were also included. For a Response control trial, when a circle was shown, the participant had to press the Response button, but the outcome was shown as 0 points. For a No Response control trial, when a square was shown, the participant had to not press the Response button, and the outcome was shown as 0 points. One control trial of each type occurred in every block of 10 trials. One hundred trials of the task were run for each participant. Of these, 9 were reversal trials, 35 were Go trials, 35 were NoGo trials, 10 were Response control trials, and 10 were NoResponse control trials. A new trial was started every 10 s, after waiting for the next scanner pulse which had a TR of 2.5 s. The rules of the task were not described to the participants, who learned the task in practice trials.

### Imaging Procedures

The fMRI neuroimaging was performed on a 3-T Siemens Prisma at the Zhangjiang International Brain Imaging Centre, Fudan University, using a 32-channel head coil. The acquisition parameters were modeled on prior investigations that aimed to minimize signal loss and distortion in the orbitofrontal cortex, anterior cingulate cortex, and amygdala (Deichmann et al. 2002; Deichmann et al. 2003; Rolls et al. 2015b). After extensive optimization procedures, we found that imaging in approximately the plane of the Sylvian fissure met the requirements for high signal-to-noise ratio in these brain regions. The details were as follows: imaging plane −30° axial; phase A>>P; TE = 25 ms; TR = 2500 ms; FOV = 208 mm, flip angle = 52°, 2 mm “pixel spacing” in 104 × 104 image (in plane pixel size 2 × 2 mm); 2.00 mm slice thickness; 72 slices; prescan normalize option; echo spacing 0.53 ms, pixel bandwidth 2290 Hz/Px; and 405 measurements. Structural scans were acquired using a T1-weighted MPRAGE sequence in a sagittal orientation, FOV = 224, 1 × 1 × 1 mm voxels, TR = 2500 ms, TE = 2.43 ms, TI = 1100 ms, and FA 7°.

### Preprocessing

The preprocessing of both functional and structural MRI data was performed using fMRIPrep 1.5.8 (Esteban et al. 2019) (https://fmriprep.readthedocs.io/en/stable/), which is based on Nipype 1.4.1 (Gorgolewski et al. 2011; Esteban et al. 2020), and is described in detail in the Supplementary Material.

### Participants

The participants were graduate students at Fudan University, Shanghai, aged between 20 and 30 (mean 23.8). Ethical permission was obtained for the study from the Research Ethics Committee of Fudan University (ref BE1944) and was performed in accordance with the Declaration of Helsinki, written information about the study was provided to participants before any scanning, and informed written consent was provided by all participants.

Neuroimaging data were available for 21 participants (9 females) who completed the Go-NoGo visual discrimination reversal task with good performance in the scanner as shown by repeated one-trial reversals and winning more than 200 points. The data from 3 other participants were not included in the analysis as their performance was below these criteria.

### fMRI Data Analysis

SPM12 was used to analyze the data. The analysis period started at the beginning of the 4-s outcome phase of each trial, the duration for the analysis was set to 2 s, and the standard hemodynamic response function was used. The subject-level SPM model included each of the five trial types, Go, NoGo, Reversal, Response Control, and No Response control. Contrasts between the activations of the different trial types were made as described in the Results. At the group level, results are described where a cluster-level analysis was significant at *P* < 0.000 FWE corrected for multiple comparisons unless otherwise stated (with voxels *P* < 0.001 uncorrected), and the number of voxels *k* in the cluster is specified. All coordinates are MNI. Results were not analyzed in early visual cortical areas, as these areas are not implicated in reward-related processing and learning by lesion studies, small differences in the physical properties of visual stimuli might have produced different effects here, and neuronal activity even at the end of the ventral stream in the inferior temporal cortex does not encode stimuli in terms of their reward value (Rolls et al. 1977). The identification of different brain areas was guided by specialized resources on the cingulate cortex (Vogt 2009) and orbitofrontal cortex (Rolls 2019b), and by the automated anatomical labeling atlas (Rolls et al. 2020c).

## Results

Neuroimaging with fMRI was performed in a Go-NoGo visual discrimination one-trial reversal task illustrated in Figure 1. Data were available for 21 participants who completed the Go-NoGo visual discrimination reversal task with good performance in the scanner as shown by repeated one-trial reversals and winning more than 200 points. In more detail, of the 189 reversal trials, 181 (96%) were perfect one-trial rule-based reversals. (A perfect one trial rule based reversal trial was a trial on which after a reward stimulus had been shown but received a loss of 25 points, on the very next trial on which a previously non-rewarded stimulus was shown, it was selected for a response and 25 points were won; and on the very next trial on which a previously rewarded stimulus was shown, it was correctly not selected for a response and only 5 points were lost.) Moreover, on the 8 trials on which the reversal did not take place in one trial, it did take place in two trials. A total of 98.6% of the Go trials were correct, 95.8% of the NoGo trials were correct, and only correct Go and NoGo trials were included in the analyses.

The activations on reversal trials were the main point of interest, and we start with these to identify the parts of the human brain involved in reward reversal. Activations related to reward (winning points) and to loss (losing points) are then described, to identify the parts of the human brain involved in reward and loss.

### Activations Related to One-Trial Reversal

Activations related to one-trial reward reversal were measured by the contrast *Reversal Trials > Go Trials*. The response is the same on both trial types, and the difference is that on the reversal trial the outcome is −25 points, and the participant must detect this lack of an expected reward, and change the rule to reverse knowledge held in memory about which stimulus is currently rewarded. The coordinates and statistics for the activations found are set out in Table 1.

**Table 1:** Activations in the one-trial reward reversal task

Contrast	Brain region	*X*	*Y*	*Z*	Number of voxels	*P* (FWE cluster)	*t* (df = 20)
**Reversal** (Reversal trials > Go trials)	Lateral orbitofrontal cortex/inferior frontal gyrus	32	64	−8	973	<0.000	6.88
	Supracallosal anterior cingulate cortex	−4	14	50	2084	<0.000	8.56
	Anterior insula	−36	18	−4	681	<0.000	9.68
	Inferior parietal cortex	38	−50	44	14 797	<0.000	13.46
**Reversal** (Reversal trials > Response control	Lateral orbitofrontal cortex/inferior frontal gyrus	36	62	4	1161	<0.000	5.98
	Supracallosal anterior cingulate cortex	6	26	26	1857	<0.000	7.02
	Anterior insula	32	24	−6	3319	<0.000	9.11
	Inferior parietal cortex	40	52	−46	2168	<0.000	10.61
**Reward** (Go trials > Response control)	Mid-orbitofrontal cortex	30	50	−12	226	0.018	5.50
**Reward** (Go trials > NoGo trials)	Ventromedial prefrontal cortex/medial orbitofrontal	8	44	−14	187	<0.000	6.18
	Pregenual anterior cingulate cortex	0	54	6	116	0.003	5.73
**Reward** (Correlation with Reward value)	Ventromedial prefrontal cortex/medial orbitofrontal	6	46	−8	173	<0.000	5.03
	Ventral striatum	8	0	−6	103	0.01	7.51
**Loss** (NoGo trials > No Response control trials)	Supracallosal anterior cingulate cortex	−8	32	38	528	0.001	6.54
	Inferior parietal cortex	44	−44	40	2220	<0.000	8.92
**Loss** (Correlation with Loss value)	Supracallosal anterior cingulate cortex/SMA	−2	14	48	1688	<0.000	6.91
	Anterior insula	32	26	4	181	<0.000	10.05
	Parietal cortex	−42	−56	54	9633	<0.000	10.19

Note: MNI coordinates are shown as X, Y, Z.

**Figure 2:**
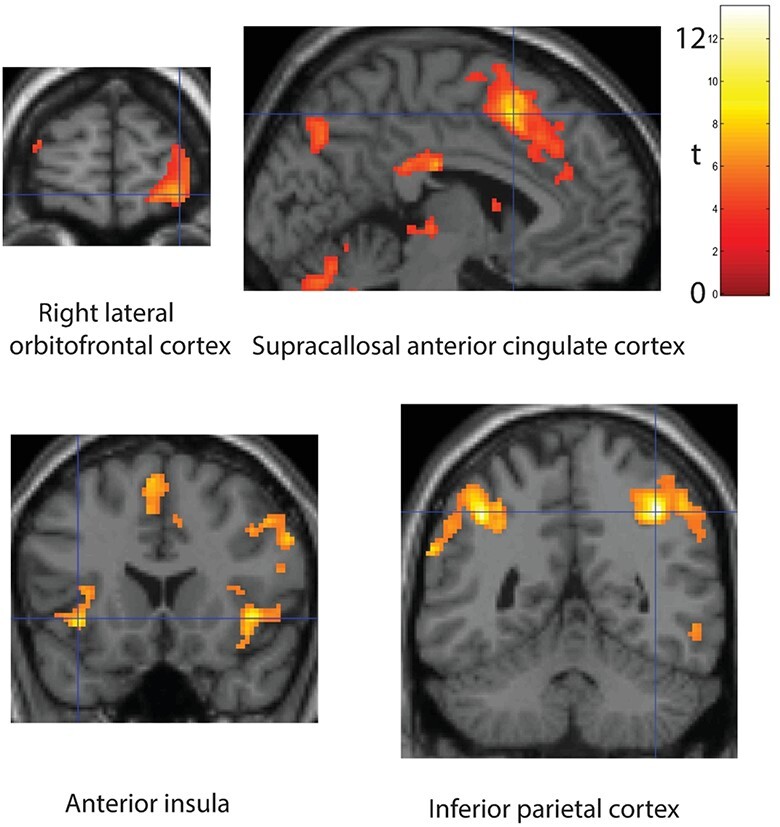
Brain regions activated on reversal trials. Activations related to one-trial reversal were measured by the contrast Reversal Trials > Go Trials. The results for the regions at the cross-hairs were significant as shown by cluster-level FWE correction with *P* < 0.000, with the coordinates provided in the text and in Table 1. The color bar shows the t value (df = 20). Images are thresholded unless otherwise stated at FWE *P* < 0.05. The anterior insula and inferior parietal images were thresholded at *P* < 0.00001 to show the extent. The orbitofrontal cortex region activated is the right lateral orbitofrontal cortex and adjoining part of the inferior frontal gyrus.

The right lateral orbitofrontal cortex/orbital and nearby part of the inferior frontal gyrus was activated by this contrast ([32 64–8] cluster FWE *P* < 0.000, number of voxels in the cluster *k* = 872), as shown in Figure 2. The activation was much greater in the right than in the left hemisphere as illustrated in Figure 2. The main region of activation is BA12 (sometimes known as area 47 or 12/47).

The supracallosal anterior cingulate cortex was also activated ([−4 14 50] FWE *P* < 0.000, *k* = 2084), as shown in Figure 2, and this was bilateral. The main region of activation is BA32 (Vogt 2009).

The anterior insula was also activated as shown in Figure 2 ([−36 18–4] FWE *P* < 0.000, *k* = 681), and this was bilateral.

An activation was also found bilaterally in the inferior parietal cortex (Figure 2, [38–50 44] FWE *P* < 0.000, *k* = 14 797), in an area known to be connected to the lateral orbitofrontal cortex and adjoining inferior frontal gyrus (Hsu et al. 2020), and this is considered in the Discussion.

These activations were also evident in the contrast *Reversal Trials > Response Control Trials*, as follows and as shown in Table 1: lateral orbitofrontal cortex/inferior frontal gyrus ([36 62 4] FWE *P* < 0.000, *k* = 1161); supracallosal anterior cingulate cortex ([6 26 26] FWE *P* < 0.000, *k* = 1857); anterior insula ([32 24–6] FWE *P* < 0.000); and (mainly right) inferior parietal cortex ([40–52 46] FWE *P* < 0.000, *k* = 2168). The point difference for this contrast is −25 vs 0, compared to −25 vs +25 in the first contrast described, so this provides useful additional evidence that reversal activates these areas, and that the reversal effect is not just when compared to a large reward.

Following a suggestion, we confirmed that the same effects were evident if the contrast was for the 9 reversal trials for each participant > the 9 immediately preceding Go trials. For example, for the lateral orbitofrontal cortex/inferior frontal gyrus, the results were [40 62 0] FWE *P* < 0.000, *k* = 254, and the effects for the other areas described above were also significant at *P* < 0.000 FWE.

### Activations Related to Reward

**Figure 3:**
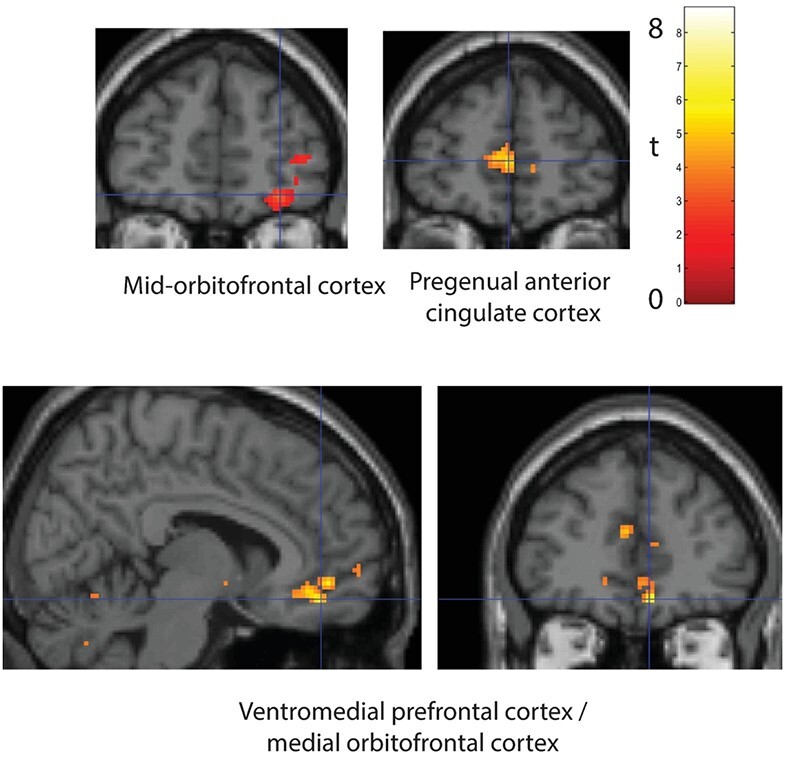
Brain regions activated by reward. Activations related to reward value were measured by the contrast Go trials (on which 25 points were won) > either Response Control trials (on which 0 points were lost) or NoGo trials (on which 5 points were lost) (see Table 1 and text). The results for the regions at the cross-hairs were significant as shown by cluster-level FWE correction, with the coordinates provided in the text and in Table 1. Conventions as in Figure 2.

Activations related to reward were assessed by the contrast *Go trials* (on which 25 points were won) > *Response control trials* on which 0 points were won. As illustrated in Figure 3 and shown in Table 1, activations were found in the mid-orbitofrontal cortex ([30 50 −12] FWE *P* < 0.018, *k* = 226, *t* = 5.5) in BA11.

This analysis was supplemented by the contrast *Go trials* (on which 25 points were won) > *NoGo trials* (on which 5 points were lost), which is a larger difference in reward value. As illustrated in Figure 3 and shown in Table 1, activations were found in the ventromedial prefrontal cortex/medial orbitofrontal cortex ([8 44 −14] FWE, *P* < 0.000, *k* = 187), and pregenual anterior cingulate cortex ([0 54 6] FWE *P* < 0.003, *k* = 116). Consistent results were found when the outcome value on every trial was used as a parametric regressor, with this analysis showing that activations in the brain regions just described were significantly correlated with reward outcome value, as shown in Table 1.

### Activations Related to Loss

**Figure 4:**
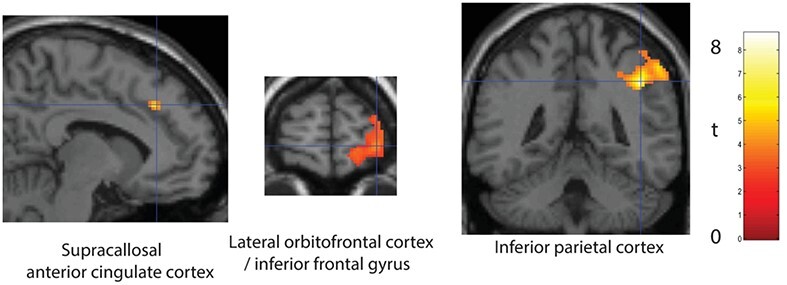
Brain regions activated by loss. Activations related to loss were measured by the contrast NoGo trials (on which 5 points were lost) > NoResponse Control trials (on which 0 points were lost). The results for the regions at the cross-hairs were significant as described in the text and in Table 1. Images are thresholded unless otherwise stated at FWE *P* < 0.05. The image for the lateral orbitofrontal cortex was thresholded at *P* < 0.01 to show the extent of the activations. Conventions as in Figure 2.

Activations related to losing points were assessed by the contrast *NoGo trials* (on which 5 points were lost) > *NoResponse Control trials* (on which 0 points were lost). On both trial types, no response was made. Although this was a small difference in loss, activations were found in the supracallosal anterior cingulate cortex ([−8 32 38] FWE *P* = 0.001, *k* = 528), and in the right inferior parietal cortex ([44 −44 40] FWE < 0.000, *k* = 2220) as illustrated in Figure 4 and shown in Table 1. Consistent results were found when the outcome value on every trial was used as a parametric regressor, with this analysis showing that activations in the brain regions just described were significantly correlated with the loss outcome value, as shown in Table 1. In addition, we examined the lateral orbitofrontal cortex/inferior frontal gyrus region activated in reversal (see Fig. 2), to measure whether that region responded to loss. Some activation to loss was found in the contrast *NoGo trials* > *NoResponse Control trials* in the right lateral orbitofrontal cortex/inferior frontal gyrus as shown in Figure 4 ([32 64 −2], *t* = 4.22, *P* = 0.0002 uncorrected), but the activation was smaller than that during reversal and was not included in Table 1 as it was not significant with brain-wide statistics using FWE correction.

## Discussion

The finding that reversal trials in one-trial rule-based visual reward reversal activate the right lateral orbitofrontal cortex and adjoining right inferior frontal gyrus including its orbital part (Fig. 2) is an important discovery in several ways.

First, it provides evidence that it is this part of the orbitofrontal cortex, area 12 *and the adjoining inferior frontal gyrus*, that is related to changing behavior on a single trial when rewards are suddenly not received. It is consistent that many aversive stimuli that should cause behavior to change, including unpleasant odors (Rolls et al. 2003a), losing money (O'Doherty et al. 2001), and many other subjectively unpleasant stimuli (Grabenhorst and Rolls 2011), activate this lateral orbitofrontal cortex region (Rolls 2019b; Rolls et al. 2020b; Rolls 2021a). In the present investigation, the activation during reversal was large (Fig. 2), with some relation to loss in the same brain region (Fig. 4). The activation of the lateral orbitofrontal cortex to non-reward in this rule-based deterministic (i.e., non-probabilistic) reversal task study is probably necessary for the reversal, in that damage to the human orbitofrontal cortex impairs an almost identical one-trial deterministic reward reversal: the patients do not change their behavior when non-reward is received on a reward-reversal trial (Rolls et al. 1994).

Second, Figure 2 shows that the activation during reversal is more in the right than the left orbitofrontal cortex and indeed that the activation extends into the right inferior frontal gyrus. It is suggested that this is because the left inferior frontal gyrus areas BA45 and BA44 which include Broca’s area are devoted to language (and especially speech production), whereas on the right without commitment to language, there is the opportunity for an enlarged right lateral orbitofrontal cortex area 12 to expand round the inferior prefrontal convexity and to utilize the inferior frontal gyrus as a route to output to premotor cortical areas for the lateral orbitofrontal cortex (Rolls 2019b; Hsu et al. 2020; Rolls et al. 2020a; Du et al. 2020b; Rolls 2021a).

Third, given that the lateral orbitofrontal cortex is involved in responses to non-rewarding, subjectively unpleasant stimuli and that if no action is possible this can lead to sadness (Rolls 2014, 2018), it has been proposed that the lateral orbitofrontal cortex is a key brain region involved in sad emotions and depression (Rolls 2016c; Rolls 2017; Rolls 2018). The discovery described here that the lateral orbitofrontal cortex is activated when behavior must change very rapidly because of non-reward is consistent with this theory of depression, by providing new evidence that the rule-based method of changing behavior in response to non-reward, which may be especially important in social interactions, does involve the lateral orbitofrontal cortex. Even more interesting, there are many studies showing that it is especially the right lateral orbitofrontal cortex that in depression has increased functional connectivity with areas such as the precuneus, posterior cingulate cortex, and temporal lobe (Cheng et al. 2016; Cheng et al. 2018a; Cheng et al. 2018b; Cheng et al. 2018c; Rolls et al. 2020a; Rolls et al. 2020b). The finding reported here provides interesting evidence relevant to the theory of depression (Rolls 2016c; Rolls 2017; Rolls 2018), by drawing attention to in particular the right lateral orbitofrontal cortex and adjoining inferior frontal gyrus as being involved in responding to non-reward to change behavior. The orbitofrontal cortex neurons that respond to non-reward in reversal can keep firing for many seconds (Thorpe et al. 1979), and that is part of the evidence that the lateral orbitofrontal contains an attractor network for non-reward (Rolls 2016c). In the theory of depression, the lateral orbitofrontal cortex attractor network is hypothesized to be more sensitive to non-reward (Rolls 2016c), and there is evidence to show that the human lateral orbitofrontal cortex does respond more to losing points in people with depressive symptoms (Xie et al. (2020) and that TMS (transcranial magnetic stimulation) of the right orbitofrontal cortex may ameliorate depression (Feffer et al. 2018).

Fourth, this investigation highlights the importance of the orbitofrontal cortex for changing behavior when rewards are not received that goes beyond what can be accounted for by model-free reinforcement learning. The key point is that when a reward is not received, the very next time that the other stimulus is shown that has recently been associated with punishment, that stimulus is selected. That requires a model with a rule that if a selection has been made, and is not rewarded, then on the very next trial the previously non-rewarded stimulus should be selected. This is a key component of social and emotional behavior in humans: that in for example social situations, if there is a hint of non-reward, perhaps a frown, then behavior can change and switch immediately. Further, we note that very rapid (in as little as one trial) switching of behavior in a deterministic (non-probabilistic) reversal task is impaired in human patients with damage to the orbitofrontal cortex (Rolls et al. 1994). Although reinforcement learning has been applied to understand optimization of rewarded behavior by the orbitofrontal cortex and areas to which it projects (Hampton et al. 2006; Hare et al. 2008; Meder et al. 2017; O'Doherty et al. 2017; Dunsmoor et al. 2019), model-free reinforcement learning cannot account for the one-trial choice of a stimulus that previously had a recent reinforcement history of being associated with punishment. If the reinforcement learning algorithm was provided with a model of one-trial rule-based reversal learning, then it could be applied, but that just shows that model-free reinforcement learning cannot itself account for one-trial reversal. (A further reason for not applying reinforcement learning analyses to the data obtained in the scanner is that the participants had been pretrained in the one-trial reward reversal task and performed that task with almost no errors. There was thus in a sense no learning to be analyzed by a reinforcement learning approach).

A model that does account for one-trial rule-based reversal learning uses an attractor network that holds the current rule online (Deco and Rolls 2005; Rolls and Deco 2016), and that ability, to hold a rule about a reward online and to use it to switch whether stimuli are treated as rewards or punishers, may be a key function supported by the highly developed primate including human orbitofrontal cortex (Rolls 2019b). In this context, the cerebral cortex is set up with local recurrent collateral connections that provide the architecture for attractor networks (Rolls 2016a, 2021a). Also in this context, the primate including the human orbitofrontal cortex is very greatly developed compared to what is present in rodents (Preuss 1995; Wise 2008; Passingham and Wise 2012; Rolls 2019b; Rolls et al. 2020b; Rolls 2021a), and indeed rodents are not known to be able to perform one-trial rule-based reward reversal (Boulougouris et al. 2007; Hervig et al. 2020). We note that this reward reversal network (Deco and Rolls 2005; Rolls and Deco 2016) is biologically plausible with integrate-and-fire attractor networks that can be reset by non-reward to switch the rule network (Rolls and Deco 2016), which then biases the mapping from the stimuli to the reward value (Deco and Rolls 2005), so is much more biologically detailed than setting up an abstract “state-space” model in which the “state” is switched by Q-learning (Wilson et al. 2014).

Figure 2 shows that the supracallosal anterior cingulate cortex and anterior insula are also activated during rule-based reversal. Their activation can be understood as follows. The orbitofrontal cortex is the brain area in primates including humans that receives from the ends of all the cortical processing systems for taste, olfactory, touch, visual, and auditory stimuli and that converts their sensory/perceptual representations into reward/punishment value-based representations. The orbitofrontal cortex thus provides a representation of the reward value of stimuli (Rolls 2019b, 2019c, 2021a). The orbitofrontal cortex then projects to the anterior cingulate cortex, as shown by anatomical studies in macaques (Ongür and Price 2000; Price 2007), and as supported by functional connectivity (Du et al. 2020b) and tract-tracing (Hsu et al. 2020) studies in humans. The cingulate cortex is implicated in action-to-reward-outcome learning, with information about actions received in the posterior cingulate cortex from the parietal cortex, and information about outcomes received in the anterior cingulate cortex from the orbitofrontal cortex (Rolls 2019a). There is now evidence in humans that the reward-related medial orbitofrontal cortex projects to the pregenual anterior cingulate cortex and that the non-reward-related lateral orbitofrontal cortex and adjoining inferior frontal gyrus project to the supracallosal anterior cingulate cortex (Hsu et al. 2020; Du et al. 2020a). The supracallosal anterior cingulate cortex area activated here by reward reversal (Fig. 2) can thus be conceived as the cingulate area that receives non-reward outcome information, and utilizes this to change actions to stimuli (Rolls 2019a, 2021a).

The anterior insular cortex area activated by reward reversal (Fig. 2) and by loss (Table 1) is a part of the insular cortex with major connections with the orbitofrontal cortex (Baylis et al. 1995; Carmichael and Price 1996; Ongür and Price 2000) and may be involved in autonomic output (Critchley and Harrison 2013; Rolls 2016b; Hassanpour et al. 2018; Rolls 2019b, 2021b), which is likely to be generated by not receiving expected rewards and by loss.

The area of the inferior parietal lobule activated in reward reversal interestingly has direct connections with the lateral orbitofrontal cortex and adjacent inferior frontal gyrus (AAL2 areas IFGorb and IFGtri (Rolls et al. 2015a)), but not with the medial orbitofrontal cortex (Hsu et al. 2020). Consistent with this and with the greater activation of the right than the left lateral orbitofrontal cortex area 12 in reversal, the right inferior frontal gyrus tends to be more strongly activated than the left, though the parietal activations for some comparisons are bilateral. Also very interestingly, the same parietal region is also activated on Loss but not on Reward trials (Table 1). Fronto-parietal systems may be useful in short-term memory and related functions, and one-trial reversal does require a short-term memory to hold the current rule in mind. In primates, some parietal cortex neurons reflect the reward value of actions (Platt and Glimcher 1999), but we show here that in humans it is especially reversal and loss that produced the parietal activation that we describe. Given the evidence on the connections of the human lateral orbitofrontal cortex with the parietal cortex (Hsu et al. 2020), and the evidence on the roles of the orbitofrontal cortex in reward, non-reward, and punishment (Rolls 2019b, 2019c), we suggest that the source of the parietal value-related activation is the orbitofrontal cortex. Beyond that, a frontoparietal system has been described as a “multiple demand” network because its activity increases as the task becomes more difficult (Shashidhara et al. 2019), but the new evidence presented here is that this parietal system is especially connected with lateral orbitofrontal cortex systems involved in reversal when an expected reward is not received and in loss more than reward. Thus part of the activity in this frontoparietal system may be related to the unpleasant effects when task demands increase, for example when non-reward or loss is detected by the lateral orbitofrontal cortex. The focus here is on the orbitofrontal cortex, for damage here is known to impair reward-related behavior, reward reversal, and emotion (Rolls 2019b), whereas similar impairments are not associated with damage to the parietal cortex (Rolls 2021a).

This study also provided evidence that in the same individuals, a different part of the orbitofrontal cortex, the medial orbitofrontal/ventromedial prefrontal cortex, is involved in representing reward value (Fig. 3). That is consistent with a great deal of previous evidence (Rolls 2019b), but it is reassuring to see this in the same study that implicates the lateral orbitofrontal cortex in one-trial rule-based reward reversal learning. Similarly, it is reassuring to see the pregenual cingulate cortex also activated by reward value, for this part of the anterior cingulate cortex receives from the medial orbitofrontal cortex (Rolls et al. 2019; Hsu et al. 2020; Du et al. 2020a) and is suggested to provide the reward outcome information for action-outcome learning by the cingulate cortex (Rolls 2019a).

In terms of the neuronal activity that may underlie the activations described here, we can relate these to neuronal recordings in macaques in a very similar task performed for juice reward. For the reversal-related activation described here (Fig. 2), this is likely to relate at least in part to orbitofrontal cortex “non-reward” neurons that respond when the reward outcome is less than expected (Rosenkilde et al. 1981; Thorpe et al. 1983). These neurons are distinct from other neurons that respond to expected loss or punishment and from others that respond to expected reward. There are also neurons that respond to reward and punishment outcomes, such as aversive or rewarding tastes, textures, and odors (Rolls et al. 1990; Rolls et al. 1996; Critchley and Rolls 1996b; Rolls et al. 2003b; Kadohisa et al. 2005; Rolls 2019b). Indeed, for the reward-related activation described here (Fig. 3), this is likely to relate to orbitofrontal cortex “reward value” neurons, examples of which in macaques are neurons that respond to rewarding visual stimuli, tastes, and odors (Rolls et al. 1989; Rolls et al. 1990; Rolls et al. 1996; Critchley and Rolls 1996a, 1996b; Rolls et al. 2003b; Kadohisa et al. 2005; Padoa-Schioppa and Assad 2008; Rolls 2019b).

In conclusion, this investigation has shown that the human right lateral orbitofrontal cortex and adjoining inferior frontal gyrus is involved in one-trial rule-based reversal, and this provides strong support for the theory that the human lateral orbitofrontal cortex is involved in changing behavior to non-reward using very rapid, rule-based, one-trial non-associative learning. This advance was made possible by the one-trial reward reversal task used here. Moreover, this casts new light on the brain mechanisms involved in reward and emotion, for it goes beyond model-free reinforcement learning (Schultz 2016; O'Doherty et al. 2017; Schultz 2017), which cannot account for the rapid non-associative change to reward selection that is described here. This discovery also provides new evidence relevant to the theory that the lateral orbitofrontal cortex is a key brain region in depression (Rolls 2016c, 2018; Rolls et al. 2020b), by showing that it is especially the right lateral orbitofrontal cortex and adjoining part of the right inferior frontal gyrus that is implicated in the effects of non-reward in humans. Indeed, in a follow-up to this theory, it has been found that TMS of the right lateral orbitofrontal cortex may ameliorate depression (Feffer et al. 2018). The computational mechanisms by which the orbitofrontal cortex detects non-reward, and supports rule-based reversal, have been considered elsewhere (Deco and Rolls 2005; Rolls and Deco 2016; Rolls 2021a).

## Notes

ETR conceived the study, took part in the data collection and analyses, and wrote the paper. DV participated in the analysis, and in the writing of the paper. YL took part in the data collection and analysis. WC took part in writing the paper. JF took part in initiating and funding the investigation, and considered the findings. All authors approved the paper.

Standard code functions available in Matlab and SPM12 were used for the analysis. fMRI datasets are available from the lead author. *Conflict of Interest:* The authors declare no competing interests.

## Funding

This work was supported as follows. J. Feng is supported by the Shanghai Science and Technology Innovation Plan (No. 15JC1400101 and No. 16JC1420402), the National Natural Science Foundation of China (No. 71661167002 and No. 91630314), The 111 Project (No. B18015), Shanghai Municipal Science and Technology Major Project (No. 2018SHZDZX01), and ZJLab. W. Cheng is supported by grants from the National Natural Sciences Foundation of China (No. 81701773, 11771010) and is sponsored by the Shanghai Sailing Program (No. 17YF1426200). W. Cheng is also sponsored by the Natural Science Foundation of Shanghai (No. 18ZR1404400). D. Vatansever is supported by a grant from the National Natural Sciences Foundation of China (No. 31950410541).

## Supplementary Material

RewardReversalSuppMat11Nov20_tgaa087
