# Supplementary material for: Rapid Rule-Based Reward Reversal and the Lateral Orbitofrontal Cortex
Source: Cereb Cortex Commun. 2020 Nov 17;1(1):tgaa087. doi: 10.1093/texcom/tgaa087 (PMC8152898; doi:10.1093/texcom/tgaa087)
Supplement: RewardReversalSuppMat11Nov20_tgaa087 [file rewardreversalsuppmat11nov20_tgaa087.docx]

**Rapid rule-based reward reversal and the lateral orbitofrontal cortex**

**Supplementary Material**

Edmund T. Rolls^1,2,3^, Deniz Vatansever^1^, Yuzhu Li^1^, Wei Cheng^1^, and Jianfeng Feng^1,3^

**Cerebral Cortex Communications (2020)**

1. Institute of Science and Technology for Brain-inspired Intelligence, Fudan University, Shanghai, 200433, China

2. Oxford Centre for Computational Neuroscience, Oxford, UK

3. Department of Computer Science, University of Warwick, Coventry CV4 7AL, UK

**Preprocessing of the MRI data**

The preprocessing of both functional and structural MRI data was performed using fMRIPrep 1.5.8 (Esteban et al. 2019) (<https://fmriprep.readthedocs.io/en/stable/>), which is based on Nipype 1.4.1 (Gorgolewski et al. 2011; Esteban et al. 2020).

*Anatomical MRI data preprocessing*

A total of two T1-weighted (T1w) 3D images were found within the input Brain Imaging Data Structure (BIDS) dataset, one of which was the original T1w image while the other was the T1w image after neck removal. Both images were corrected for intensity non-uniformity (INU) with N4BiasFieldCorrection (Tustison et al. 2010), distributed with ANTs 2.2.0 (Avants et al. 2008). The T1w-reference was then skull-stripped with a Nipype implementation of the antsBrainExtraction.sh workflow (from ANTs), using OASIS30ANTs as the target template. Brain tissue segmentation of cerebrospinal fluid (CSF), white-matter (WM) and gray-matter (GM) was performed on the brain-extracted T1w image using fast (FSL 5.0.9) (Zhang et al. 2001). A T1w-reference map was computed after registration of the two T1w images (after INU-correction) using mri_robust_template (FreeSurfer 6.0.1) (Reuter et al. 2010). Brain surfaces were reconstructed using recon-all (FreeSurfer 6.0.1) (Dale et al. 1999), and the brain mask that was estimated previously was refined with a custom variation of the method to reconcile ANTs-derived and FreeSurfer-derived segmentations of the cortical gray-matter of Mindboggle (Klein et al. 2017). Volume-based spatial normalization to two standard spaces (MNI152NLin6Asym, MNI152NLin2009cAsym) was performed through nonlinear registration with antsRegistration (ANTs 2.2.0), using brain-extracted versions of both the T1w reference and the T1w template. The following templates were selected for spatial normalization: FSL’s MNI ICBM 152 non-linear 6th Generation Asymmetric Average Brain Stereotaxic Registration Model [(Evans et al. 2012), TemplateFlow ID: MNI152NLin6Asym], ICBM 152 Nonlinear Asymmetrical template version 2009c [(Fonov et al. 2011), TemplateFlow ID: MNI152NLin2009cAsym].

*Functional MRI data preprocessing*

For each BOLD run per subject, the following preprocessing was performed. First, a reference volume and its skull-stripped version were generated using a custom methodology of fMRIPrep. A B0-nonuniformity map (or fieldmap) was estimated based on a phase-difference map calculated with a dual-echo GRE (gradient-recall echo) sequence, processed with a custom workflow of SDCFlows inspired by the epidewarp.fsl script and further improvements in the Human Connectome Project (HCP) Pipelines (Glasser et al. 2013). A deformation field to correct for susceptibility distortions was estimated based on the fMRIPrep’s fieldmap-less approach. The deformation field is that resulting from co-registering the BOLD reference to the same-subject’s T1w-reference with its intensity inverted (Huntenburg 2014; Wang et al. 2017). Registration is performed with antsRegistration (ANTs 2.2.0), and the process regularized by constraining deformation to be nonzero only along the phase-encoding direction, and modulated with an average fieldmap template (Treiber et al. 2016). The fieldmap was then co-registered to the target EPI (echo-planar imaging) reference run and converted to a displacement field map (amenable to registration tools such as ANTs) with FSL’s fugue and other SDCflows tools. Based on the estimated susceptibility distortion, a corrected EPI reference was calculated for a more accurate co-registration with the anatomical reference. The BOLD reference was then co-registered to the T1w reference using bbregister (FreeSurfer) which implements boundary-based registration (Greve and Fischl 2009). Co-registration was configured with six degrees of freedom. Head-motion parameters with respect to the BOLD reference (transformation matrices, and six corresponding rotation and translation parameters) were estimated before any spatiotemporal filtering using mcflirt (Jenkinson et al. 2002) (FSL 5.0.9). BOLD runs were slice-time corrected using 3dTshift from AFNI 20160207 (Cox and Hyde 1997). The BOLD time-series (including slice-timing correction when applied) were resampled onto their original, native space by applying a single, composite transform to correct for head-motion and susceptibility distortions. These resampled BOLD time-series will be referred to as preprocessed BOLD in original space, or just preprocessed BOLD. The BOLD time-series were resampled into several standard spaces, correspondingly generating the following spatially-normalized, preprocessed BOLD runs: MNI152NLin6Asym, MNI152NLin2009cAsym. First, a reference volume and its skull-stripped version were generated using a custom methodology of fMRIPrep. Automatic removal of motion artifacts using independent component analysis (ICA-AROMA) (Pruim et al. 2015) was performed on the preprocessed BOLD on MNI space time-series after removal of non-steady state volumes and spatial smoothing with an isotropic, Gaussian kernel of 6mm FWHM (full-width half-maximum). Corresponding “non-aggresively” denoised runs were produced after such smoothing. Additionally, the “aggressive” noise-regressors were collected and placed in the corresponding confounds file. Several confounding time-series were calculated based on the preprocessed BOLD: framewise displacement (FD), DVARS and three region-wise global signals. FD and DVARS were calculated for each functional run, both using their implementations in Nipype and following the definitions by Power et al. (2014). The three global signals were extracted within the CSF, the WM, and the whole-brain masks. Additionally, a set of physiological regressors were extracted to allow for component-based noise correction (CompCor) (Behzadi et al. 2007). Principal components were estimated after high-pass filtering the preprocessed BOLD time-series (using a discrete cosine filter with 128s cut-off) for the two CompCor variants: temporal (tCompCor) and anatomical (aCompCor). tCompCor components were then calculated from the top 5% variable voxels within a mask covering the subcortical regions. This subcortical mask was obtained by heavily eroding the brain mask, which ensures it does not include cortical GM regions. For aCompCor, components were calculated within the intersection of the aforementioned mask and the union of CSF and WM masks calculated in T1w space, after their projection to the native space of each functional run (using the inverse BOLD-to-T1w transformation). Components were also calculated separately within the WM and CSF masks. For each CompCor decomposition, the k components with the largest singular values were retained, such that the retained components’ time series were sufficient to explain 50 percent of variance across the nuisance mask (CSF, WM, combined, or temporal). The remaining components were dropped from consideration. The head-motion estimates calculated in the correction step were also placed within the corresponding confounds file. The confound time series derived from head motion estimates and global signals were expanded with the inclusion of temporal derivatives and quadratic terms for each (Satterthwaite et al. 2013). Frames that exceeded a threshold of 0.5 mm FD or 1.5 standardised DVARS were annotated as motion outliers. All resamplings can be performed with a single interpolation step by composing all the pertinent transformations (i.e. head-motion transform matrices, susceptibility distortion correction when available, and co-registrations to anatomical and output spaces). Gridded (volumetric) resamplings were performed using antsApplyTransforms (ANTs), configured with Lanczos interpolation to minimize the smoothing effects of other kernels (Lanczos 1964). Non-gridded (surface) resamplings were performed using mri_vol2surf (FreeSurfer). Many internal operations of fMRIPrep use Nilearn 0.6.1 (Abraham et al. 2014), mostly within the functional processing workflow. For more details of the pipeline, see the section corresponding to workflows in fMRIPrep’s documentation.

The preprocessed BOLD timeseries in MNI152NLin6Asym space (2mm isotropic resolution) were run through a smoothing procedure using SPM12 with a Gaussian kernel of 6 mm FWHM (full-width half-maximum) and were employed in the main fMRI data analysis. In the modelling of task fMRI data, the global signals from WM, CSF as well as the six head motion parameters were included as nuisance variables.

References

Abraham A, Pedregosa F, Eickenberg M, Gervais P, Mueller A, Kossaifi J, Gramfort A, Thirion B, Varoquaux G. 2014. Machine learning for neuroimaging with scikit-learn. Front Neuroinform 8:14.

Avants BB, Epstein CL, Grossman M, Gee JC. 2008. Symmetric diffeomorphic image registration with cross-correlation: evaluating automated labeling of elderly and neurodegenerative brain. Med Image Anal 12:26-41.

Behzadi Y, Restom K, Liau J, Liu TT. 2007. A component based noise correction method (CompCor) for BOLD and perfusion based fMRI. Neuroimage 37:90-101.

Cox RW, Hyde JS. 1997. Software tools for analysis and visualization of fMRI data. NMR Biomed 10:171-178.

Dale AM, Fischl B, Sereno MI. 1999. Cortical surface-based analysis. I. Segmentation and surface reconstruction. Neuroimage 9:179-194.

Esteban O, Markiewicz CJ, Blair RW, Moodie CA, Isik AI, Erramuzpe A, Kent JD, Goncalves M, DuPre E, Snyder M, Oya H, Ghosh SS, Wright J, Durnez J, Poldrack RA, Gorgolewski KJ. 2019. fMRIPrep: a robust preprocessing pipeline for functional MRI. Nat Methods 16:111-116.

Esteban O, Markiewicz CJ, Johnson H, Ziegler E, Manhes-Savio A, Jarecka D, Burns C, Ellis DG, Hamalainen C, Notter MP, Yvernault B, Salo T, Waskom M, Goncalves M, Jordan K, Wong J, Dewey BE, Madison C, Benderoff E, Clark D, Loney F, Clark D, Keshavan A, Joseph M, Nielson DM, Dayan M, Modat M, Gramfort A, Bougacha S, Pinsard B, Berleant S, Christian H, Rokem A. 2020. nipy/nipype: 1.4.2. doi: 10.5281/ZENODO.3668316.

Evans AC, Janke AL, Collins DL, Baillet S. 2012. Brain templates and atlases. Neuroimage 62:911-922.

Fonov V, Evans AC, Botteron K, Almli CR, McKinstry RC, Collins DL, Brain Development Cooperative G. 2011. Unbiased average age-appropriate atlases for pediatric studies. Neuroimage 54:313-327.

Glasser MF, Sotiropoulos SN, Wilson JA, Coalson TS, Fischl B, Andersson JL, Xu J, Jbabdi S, Webster M, Polimeni JR, Van Essen DC, Jenkinson M, Consortium WU-MH. 2013. The minimal preprocessing pipelines for the Human Connectome Project. Neuroimage 80:105-124.

Gorgolewski K, Burns CD, Madison C, Clark D, Halchenko YO, Waskom ML, Ghosh SS. 2011. Nipype: a flexible, lightweight and extensible neuroimaging data processing framework in python. Front Neuroinform 5:13.

Greve DN, Fischl B. 2009. Accurate and robust brain image alignment using boundary-based registration. Neuroimage 48:63-72.

Huntenburg JM. 2014. Evaluating nonlinear coregistration of BOLD EPI and T1w images [master]. Berlin: Freie Universität.

Jenkinson M, Bannister P, Brady M, Smith S. 2002. Improved optimization for the robust and accurate linear registration and motion correction of brain images. Neuroimage 17:825-841.

Klein A, Ghosh SS, Bao FS, Giard J, Hame Y, Stavsky E, Lee N, Rossa B, Reuter M, Chaibub Neto E, Keshavan A. 2017. Mindboggling morphometry of human brains. PLoS Comput Biol 13:e1005350.

Lanczos C. 1964. Evaluation of Noisy Data. Journal of the Society for Industrial and Applied Mathematics Series B Numerical Analysis 1:76-85.

Power JD, Mitra A, Laumann TO, Snyder AZ, Schlaggar BL, Petersen SE. 2014. Methods to detect, characterize, and remove motion artifact in resting state fMRI. Neuroimage 84:320-341.

Pruim RHR, Mennes M, van Rooij D, Llera A, Buitelaar JK, Beckmann CF. 2015. ICA-AROMA: A robust ICA-based strategy for removing motion artifacts from fMRI data. Neuroimage 112:267-277.

Reuter M, Rosas HD, Fischl B. 2010. Highly accurate inverse consistent registration: a robust approach. Neuroimage 53:1181-1196.

Satterthwaite TD, Elliott MA, Gerraty RT, Ruparel K, Loughead J, Calkins ME, Eickhoff SB, Hakonarson H, Gur RC, Gur RE, Wolf DH. 2013. An improved framework for confound regression and filtering for control of motion artifact in the preprocessing of resting-state functional connectivity data. Neuroimage 64:240-256.

Treiber JM, White NS, Steed TC, Bartsch H, Holland D, Farid N, McDonald CR, Carter BS, Dale AM, Chen CC. 2016. Characterization and Correction of Geometric Distortions in 814 Diffusion Weighted Images. PLoS One 11:e0152472.

Tustison NJ, Avants BB, Cook PA, Zheng Y, Egan A, Yushkevich PA, Gee JC. 2010. N4ITK: improved N3 bias correction. IEEE Trans Med Imaging 29:1310-1320.

Wang S, Peterson DJ, Gatenby JC, Li W, Grabowski TJ, Madhyastha TM. 2017. Evaluation of Field Map and Nonlinear Registration Methods for Correction of Susceptibility Artifacts in Diffusion MRI. Front Neuroinform 11:17.

Zhang Y, Brady M, Smith S. 2001. Segmentation of brain MR images through a hidden Markov random field model and the expectation-maximization algorithm. IEEE Trans Med Imaging 20:45-57.
